# WU Polyomavirus (WUPyV): A Recently Detected Virus Causing Respiratory Disease?

**DOI:** 10.3390/v1030678

**Published:** 2009-11-04

**Authors:** Michael Kleines, Martin Häusler, Alexander Krüttgen, Simone Scheithauer

**Affiliations:** 1 Division of Virology, Department of Medical Microbiology, University Hospital Aachen, RWTH Aachen, Pauwelsstrasse 30, 52074 Aachen, Germany; E-Mail: akruettgen@ukaachen.de; 2 Deparment of Pediatrics, University Hospital Aachen, RWTH Aachen, Pauwelsstrasse 30, 52074 Aachen, Germany; E-Mail: haeusler@rwth-aachen.de; 3 Department of Infection Control and Infectious Diseases, University Hospital Aachen, RWTH Aachen, Pauwelsstrasse 30, 52074 Aachen, Germany; E-Mail: sscheithauer@ukaachen.de

**Keywords:** respiratory tract disease, infection, novel virus, *Polyomaviridae*, WUPyV

## Abstract

The WU polyomavirus (WUPyV) is a novel member of the family *Polyomaviridae* recently detected in respiratory tract specimens by shotgun sequencing. Intriguingly, viral genome has been detected in 0.4% to 11.5% of respiratory tract specimens from children with respiratory disease. The levels of co-infection with established respiratory viruses were in the range between 30.8% and 91.7%. Moreover, some studies report detection of WUPyV in stool or serum. So far, WUPyV infections can not be distinguished from other viral infections by means of clinical symptoms. Respiratory tract disease like pneumonia or bronchitis is frequently observed in patients harbouring WUPyV. Detection of viremia suggests systemic infections. However, the available data do not prove WUPyV to be a human pathogen. Further investigations are necessary.

## 1. Introduction

Respiratory tract infections are a major cause of hospital admission in infants and young children. They are caused by a broad spectrum of established respiratory tract pathogens, particularly by viruses including the respiratory syncytial virus (RSV), the most important cause of severe respiratory tract infections in young children, influenza viruses, parainfluenza viruses, rhinoviruses, coronaviruses (229E, OC43), and adenoviruses. Studies investigating the entire spectrum of established respiratory tract pathogens are rare and mostly based on the detection of pathogen specific antibodies. Only in recent years, a few studies became available presenting data based on the direct detection of relevant infectious agents. Creer and co-workers [1] identified at least one potential pathogen in 69% of specimens from adults suffering from lower respiratory tract infections (LRTI, 63% contained viruses, 26% contained bacteria) still leaving a significant diagnostic gap to be filled with so far unidentified pathogenic microorganisms. Additional agents associated with respiratory tract infections have been discovered recently, e.g. the human metapneumovirus (hMPV) [2], coronaviruses (NL63, HKU1) [3,4], and the human bocavirus (HBoV) [5]. Furthermore, herpes viruses may contribute to fill this gap as they are assumed to trigger respiratory tract infections [6,7]. Unfortunately, relevant studies did not often include them.

By now, the pathogenic impact of the human metapneumovirus and its relevance on respiratory tract infections has been well established. It is a common cause of upper (URTI) and lower respiratory tract infections (LRTI) in children, elderly, and immunocompromised persons; asymptomatic infections seem to be uncommon [8]. hMPV has been identified to be an important cause of bronchiolitis in young children. The novel human coronavirus HCoV-NL63 is also widely accepted to be a significant respiratory tract pathogen. A strong association with croup has been shown [9]. Human coronavirus HCoV-HKU1 infections are observed rather infrequent, often in children with underlying diseases [4]. Several reports link the virus with pneumonia and bronchiolitis. For the human bocavirus (HBoV) an increasing number of reports present evidence for an association with respiratory tract infections; however, co-infections with established respiratory tract pathogens are frequent.

Recently, the WU polyomavirus was detected in respiratory tract specimens [10].

## 2. Discovery of WU Polyomavirus (WUPyV)

The first polyomaviruses were isolated in 1971 and named JC virus and BK virus. In 2007 and 2008 3 further viruses of this particular family were described for the first time: WU polyomavirus, KI polyomavirus, and Merkel cell polyomavirus [11]. WUPyV was discovered by shot gun sequencing of nasopharyngeal aspirate of a three-years-old child from Australia suffering from pneumonia [10]. The investigators isolated total nucleic acids from the nasopharyngeal aspirate, randomly amplified fragments, and cloned them. Sequences of the obtained clones gained by shot gun sequencing were subjected to automated editing and database searches.

The combination of these technologies led to the identification of a DNA-sequence distant to all sequences included in the sequence database, but related to viruses of the family *Polyomaviridae*. Thus, the identification of a novel virus belonging to this viral family has been proposed, exclusively based on molecular data. So far, no system for *in vitro* or *in vivo* propagation has been discovered, neither a cell line nor an animal model. Thus, until now Koch’s postulates could not be fulfilled. However, the molecular data form reasonable evidence to propose the existence of the novel virus WUPyV.

Large-scale screening based technologies as the one used for the discovery of WUPyV may be a powerful tool in discovering new infectious agents causing respiratory tract diseases particularly with regard to the etiological gap of up to 40%.

## 3. Classification of WUPyV

The novel virus has a 5229 bp dsDNA genome with a GC-content of 39%. The genome contains an early coding region encoding the T-antigens, a late coding region encoding structural proteins, and a non-coding control region [10]. All features are typical for members of the polyomavirus family. The sequence identity of WUPyV at the amino acid level to all of the well-known polyomaviruses ranged from 15% – 49%. The closest relative within the virus family is the KI polyomavirus (KIPyV) with a sequence identity of 68%, which was described briefly before WUPyV. Thus, both viruses were grouped into a new subclass of the polyomavirus family. However, the international committee on taxonomy of viruses (ICTV) has not recognised WUPyV or KIPyV yet.

## 4. Molecular Features

Typical members of the family *Polyomaviridae* form small, non-enveloped, icosahedral particles with 40–45 nm in diameter containing a supercoiled dsDNA genome [11]. The virion is made up by 72 pentamers of the structural protein VP1, each pentamer binds to a single copy of VP2 or VP3. The genome is attached to cellular histones. There is no evidence hinting at a virus structure for WUPyV differing from other polyomaviruses. However, WUPyV virions have not been displayed by electron microscopy, thus there is no evidence for the precise virion structure at this stage.

Based on the genome organisation, two subclasses of the polyomavirus family have been postulated: the primate-like group and the mouse polyoma-like group [10]. The genome of members of both subclasses is divided into three regions. The early coding region encodes at least the small T-antigen and the large T-antigen. Both are binding partners of the RB-protein and p53. The late coding region encodes at least the structural proteins VP1-VP3, and the non coding control region contains viral promoters and the origin of replication. The early coding region of members of the mouse polyoma-like group additionally encodes the medium T-antigen, whereas the late coding region of members of the primate-like group additionally encodes the structural protein agnoprotein. Apart from KIPyV, the JC virus and the BK virus are the closest relatives of WUPyV based on genome organisation and amino acid sequence. The early coding region contains a splice site for the large T-antigen and binding domains for the RB-protein and p53. However, WUPyV neither encodes open reading frames for the middle T-antigen nor the agnoprotein. Thus, WUPyV and KIPyV were provisionally grouped into a third subclass of the polyomavirus family. So far, no expression studies have been conducted on WUPyV to clarify whether the predicted open reading frames are in fact expressed during the viral infection of the target. Interestingly, as shown for HBoV the predicted transcripts of a virus are not necessarily identical to the transcripts detectable in an infected cell [12]. There is the need for an appropriate experimental system to carry out expression studies on WUPyV for further characterization.

## 5. Course of Infection

Polyomaviruses have a restricted host range. The BK virus and the JC virus, the closest relatives of WUPyV within the well established members of the virus family, are human-specific. This suggests a host range restricted to humans for WUPyV, too. Infection with BKV takes place in early childhood; 90% of 5–9 years old children are seropositive. WUPyV infections have been reported predominantly in children <3 years of age [10] suggesting a strong increase of seropositivity during early childhood for this virus, too. The incubation period of WUPyV infections is unknown. Respiratory transmission has been suggested for JC virus and BK virus although the diseases related to these viruses are not involving the respiratory tract. Both viruses can be extracted from tonsil tissue [13,14]. The viral entry route for WUPyV is not completely defined. It could be defined by uptake through dividing cells of the upper respiratory tract and the oropharynx. In this case it would be likely that mostly children are at risk of infection as, due to their growing up, their anabolism is enhanced involving increased cell division. Alternatively transmission may occur occasionally by blood transfusion and organ transplantation.

JC virus and BK virus primary infections are typically subclinical or linked to mild respiratory illness [13,15]. Viral dissemination to the target cells occurs via blood [16]. Sites of lifelong persistence are epithelial cells of the kidney for BK virus [17] and lymphocytes, oligodendrocytes as well as epithelial cells of kidney for JC virus [18]. 5% of immunocompetent BK virus carriers show viruria [19], 20 – 30% show shedding of JC virus [20]. This indicates that viral persistence can be terminated and viral reactivation can take place even in immunocompetent persons. Reactivation can be assumed to an obviously higher extent in immunocompromised individuals. Concordantly the related diseases, hemorrhagic cystitis and polyomavirus nephropathy for BK virus and progressive multifocal leukencephalopathy (PML) for JC virus, predominantly occur in persons with reduced immunocompetence. As WUPyV was detected in a respiratory tract specimen the respiratory tract can be assumed to be the entry site for this virus, too. The virus was detected in serum indicating that the dissemination occurs via blood. So far, sites of persistence have not been identified. The virus has not been detected in urine [10]. Thus, epithelial cells of kidney may not be the site of persistence for WUPyV.

WUPyV is made responsible for upper and lower respiratory tract diseases, e.g. bronchiolitis, croup, and pneumonia [10]. Besides nasopharyngeal samples, WUPyV could also be detected in stool samples [21], even in patients without any apparent clinical respiratory symptoms [22]. However, it has to be determined, whether WUPyV is able to replicate in the intestine or whether it accumulates there by oral ingestion.

## 6. Detection of WUPyV-Infections

For detection of WUPyV-infections several conventional PCR formats as well as real-time PCR protocols were developed reporting sensitivities up to 100% and specificities up to 97.7% [10,23–25]. Antigen or antibody detection formats have not been reported, so far. No culture system is known at this time.

The diagnostics of established respiratory viruses is based on a broad spectrum of available assays, aiming at the detection of virus-specific antibodies, viral protein, or viral nucleic acids. Acute respiratory tract infections commonly have a short incubation period. Thus, antibodies are usually not present at the beginning of the acute phase of the disease. For this reason, detection of virus-specific antibodies by complement fixation assays, enzyme linked immunosorbent assays (ELISA), or immunofluorescence assays (IFA), all of which are available for established respiratory viruses, is inappropriate for the identification of the etiologic agent causing an acute respiratory tract infection, but useful for retrospective or epidemiological studies. Direct detection of viral protein or viral nucleic acids is the mode of choice for respiratory virus diagnostics. Viral cell culture represents the gold standard for the well established respiratory viruses, but is too slow for in time diagnostics. Less time consuming methods have been developed. These comprise polymerase chain reaction (PCR), antigen-specific ELISA, antigen-specific IFA, and, available only for a few respiratory viruses, very fast immunochromatographic assays. Sensitivity and specificity of these assays vary considerably. The PCR displays the highest sensitivity. Thus, being restricted to PCR formats for detection of WUPyV is no bias to sensitivity. Particularly real time PCR formats have an established track record in both, very sensitive detection and differentiation between colonisation and acute infection based on the quantitative data [26,27]. Availability of automated extraction of nucleic acids permits short processing times and, by this, handling of large specimen cohorts [28].

## 7. Epidemiology

Following the discovery of WUPyV in Australia, the virus was detected in specimens from patients with respiratory tract disease on all continents suggesting a worldwide distribution [10,29–31]. So far, WUPyV-DNA was reported to be found in respiratory tract specimens (e.g. nasopharyngeal washes, tracheal secretion, BAL), serum, and faeces. The virus could not be detected in urine or from UV light-associated primary malignant lymphomas [10,32]. Specimens from other malignant diseases have not been investigated. The use of tracheal secretion for diagnostics has been shown to lead to an underestimation of the rate of positive specimens compared to other respiratory materials for HBoV [33]. This may be true for WUPyV, too.

The data available are mainly based on retrospective studies exclusively including symptomatic patients. The detection rate in respiratory samples from children with respiratory disease varies from 0.4% to 11.5% [34,35]. The age of WUPyV infected patients ranged from a few weeks to 53 years, children <3 years of age were dominating. Infections were predominantly detected in late winter, spring, and early summer [36]. High infection rates were reported for study populations preselected for lack of immunocompetence. HIV positive patients had detection rates of up to 35.7% in respiratory tract specimens and 8.3% in blood [37,38]. The rates of co-infection with established respiratory viruses lay between 30.8% and 91.7% [39,40], commonly exceeding 50%. WUPyV was detectable in blood, possibly indicating its potential for systemic infections.

Le and co-workers [41] presented evidence for viral persistence, Wattier *et al.* [36] for nosocomial infections with WUPyV.

The real time protocols available allow the quantification of WUPyV. Quantification of viral loads in respiratory tract specimens revealed viral titers up to 10^10^ copies/ml, but low and medium viral loads were dominating [21,24]. No correlation between viral load and the rate of co-infection or clinical diagnoses was observed.

Few studies included asymptomatic control groups [34,36,42–44]. The results were not concordant and reached from higher detection rates in the control group to higher detection rates among the group of patients with respiratory tract diseases.

Prospective studies have been published only recently. Van der Zalm and co-workers [34] reported the detection of WUPyV in a cohort of 18 children. Their parents were contacted twice a week over a 6-months period (November to April) and asked for symptoms of respiratory tract disease in their children. Every two weeks respiratory tract specimens were collected, regardless of respiratory symptoms. 11.5% of the specimens of children with symptoms were WUPyV positive, but only 3.1% of specimens of healthy children, indicating at least an association of WUPyV with disease.

## 8. Therapy

To this time there are no studies available concerning therapeutic interventions. This is mainly due to the retrospective nature of most investigations. Further, no causal association between WUPyV and respiratory disease has been shown yet [45]. As the need for therapeutics is driven by associating infections with disease, studies on therapeutic interventions are not to be expected in the near future. For BK virus associated nephropathy in renal transplant patients, reduction of immunosuppressive therapy is recommended if possible. In the case of progressive renal dysfunction fluoroquinolones (antibacterials), intravenous immunoglobulines, leflunomide, and – in otherwise refractory cases – cidofovir could be administered. For JC virus caused progressive multifocal leukoencephalopathy (PML) no specific therapy exists. In HIV-positive patients HAART induced improvement of cellular function may lead to an at least temporary improvement. Failure of treatment approaches with interferon alfa-2b, cytarabine, cidofovir, and topotecan has been documented [46].

## 9. Conclusions

The discovery of novel respiratory viruses has the potential to diminish the diagnostic gap for respiratory tract infections. Creer and co-workers [1], who omitted the recently identified respiratory viruses in their study on adults, reported a diagnostic gap of 31%. The share of specimens from children negative for any respiratory pathogen investigated was 22% when hMPV, HBoV, and HCoV-NL63 were included [47]. No study included the novel polyomaviruses or herpes viruses until now. Thus, a further reduction of the respiratory tract infections of unknown origin seems reasonable. However, a substantial gap is remaining leaving sufficient room for additional respiratory viruses to be discovered in the future.

So far it has not been finally proven, if WUPyV is a real causative agent for respiratory diseases. Association of virus detection with previously unexplained respiratory disease led to the tempting idea of WUPyV representing a new etiologic agent, particularly in cases where no other respiratory tract pathogen could be identified. Furthermore, the association of mouse pneumotropic polyomavirus with intestinal pneumonia and significant mortality [48] indicates that polyomaviruses have the capacity to be respiratory tract pathogens.

Many studies correlating the detection of WUPyV with the incidence of respiratory symptoms argue for this hypothesis, but it remains difficult to prove as asymptomatic control groups have not been investigated sufficiently. Additionally, the studies including control groups report dissenting findings. However, in two studies asymptomatic individuals display a lower frequency of virus detection compared to symptomatic patients. One of these is the only prospective study available.

The difficulty to prove WUPyV to be a respiratory pathogen may be due to the fact that most studies available so far try to correlate the virus with respiratory tract disease in general. In case of WUPyV being the causative agent of a particular entity of respiratory tract disease, inclusion of patients displaying any kind of respiratory disease may bias the investigation. Focussing on a single entity of respiratory disease proved successful for HCoV-NL63, which was shown to cause croup.

No association of WUPyV viral loads and clinical symptoms could be observed so far, and co-infections with other viruses were described frequently. This weakens the hypothesis of WUPyV being a respiratory tract pathogen. However, collection of samples was not performed by means of a standardized protocol among the various groups or at a defined time point, after onset of symptoms. As viral loads decrease during the acute phase of infection, the combination of longitudinal data from different time points in one analysis may bias the correlation of viral loads and symptoms. Furthermore, detection of co-infection does not exclude pathogenic potential for WUPyV, as early childhood is characterized by subsequent episodes of respiratory infections. Co-detection of the declining pathogen responsible for the last episode of respiratory disease and the pathogen responsible for the present, acute disease has to be expected. Only determination of the viral load kinetics would allow defining the clinical impact of a detectable microorganism.

The large T-antigen of WUPyV contains binding sites for the RB protein and p53. Thus, tumourigenic potential cannot be excluded. However, the association of WUPyV with other diseases, particularly tumour diseases has not been sufficiently investigated yet.

Taken together, the currently available data neither prove nor deny WUPyV to be a respiratory tract pathogen. Several scenarios may describe the role of the virus best. WUPyV may be

1. part of the endogenous viral flora without pathogenic potential;
2. an opportunistic pathogen with pathogenic potential in the respiratory tract under conditions still to be defined;
3. a pacemaker for secondary infections;
4. a viral pathogen using the respiratory tract only as an entry route to reach the final target cell; infection of this cell type is the basis of a disease not related to the respiratory tract.

To reach a final conclusion more powerful, controlled studies need to be performed prospectively. However, retrospective analysis of age- and time-matched control populations also permits conclusions if the data collected at the time are related to respiratory illness. Ideally, prospective studies would be carried out in a multicenter-design including control groups concentrating on the correlation of WUPyV with single entities of respiratory tract diseases. Criteria for inclusion of specimens have to be comparable, as well as the time point and the protocol for extraction of specimens. All known or suspected respiratory tract pathogens should be included and follow-up investigations should be performed. The detection method of choice would be quantitative PCR to allow discrimination between colonisation and productive infection. Additionally, serological investigations should be included to show that the patients seroconvert in response to an infection. The study participants should be evaluated before enrolment. Beside investigation of respiratory tract disease, the cell type hosting the persisting virus has to be identified and a possible association with tumour diseases has to be investigated.
